# A Gastroenteritis Outbreak Caused by Noroviruses in Greece

**DOI:** 10.3390/ijerph8083468

**Published:** 2011-08-22

**Authors:** Apostolos Vantarakis, Kassiani Mellou, Georgia Spala, Petros Kokkinos, Yiannis Alamanos

**Affiliations:** 1 Environmental Microbiology Unit, Department of Public Health, Medical School, University of Patras, Patras 26500, Greece; E-Mails: pkokkin@med.upatras.gr (P.K.); ialamano@upatras.gr (Y.A.); 2 Hellenic Center for Disease Control and Prevention, Athens 15123, Greece; E-Mails: kmellou@gmail.com (K.M.); gspala@gmail.com (G.S.)

**Keywords:** *Noroviruses*, outbreak, Greece, phylogenetic analysis, genogroup II (GGII)

## Abstract

In June 2006, an outbreak alert regarding cases of acute gastroenteritis in a region in North Eastern Greece (population 100,882 inhabitants), triggered investigations to guide control measures. The outbreak started the first days of June, and peaked in July. A descriptive epidemiological study, a virological characterization of the viral agent identified from cases as well as a phylogenetic analysis was performed. From June 5 to September 3, 2006 (weeks 23–44), 1,640 cases of gastroenteritis (45.2% male and 54.8% female, aged 3 months to 89 years) were reported. The overall attack rate for the period was 16.3 cases/1,000 inhabitants. About 57% of cases observed were under the age of 15 years. nalysis of faecal samples identified *Norovirus GII* strains. Fifteen different *Norovirus GII* strains were recorded, presenting a homology of 94.8% (86–97%) to GII strains obtained from GenBank. The long duration of the outbreak suggests an important role of person-to-person transmission, while the emergence of the outbreak was possibly due to contaminated potable water, although no viruses were detected in any tested water samples. This outbreak underscores the need for a national surveillance system for acute non-bacterial gastroenteritis outbreaks.

## 1. Introduction

Gastrointestinal tract infections are common in both developing and developed countries [1] and are produced by a wide variety of enteropathogens, including bacteria, viruses and parasites. The main causes of viral gastroenteritis include noroviruses and rotaviruses [2–4]. Since the first described outbreak of *Norwalk* virus gastroenteritis in an elementary school in Norwalk (OH, USA) in the fall of 1968 [5], studies have shown that viral infections, especially those due to *Noroviruses* (NoVs), are the most frequent causes of acute gastroenteritis in the community [6–8]. NoVs infect all age groups, with particularly severe disease occurring in young children, the elderly and persons with chronic illnesses [9]. Because of the low infectious dose (10–100 viral particles can induce symptoms), short-lived immunity, and high stability in the environment, these viruses are especially contagious and outbreaks are characterised by high secondary attack rates [9]. NoV infections have historically been described as mild and self-limiting [6,8]. Studies in the 1970s were the basis for the criteria used by Kaplan *et al.* [10] to discern outbreaks with a viral aetiology, which include stool culture negative for bacterial pathogens, mean (or median) duration of illness 12–60 h, vomiting in 50% of cases, and, if known, mean (or median) incubation period of 24–48 h [10]. Kaplan *et al.* criteria are still considered valid [11]. NoVs cause outbreaks in a wide range of settings, especially in hotels [12], hospitals [13], retirement centres [14], schools [15,16], and cruise ships [17]. The mode of transmission may be foodborne, waterborne or person-to-person contact. Point source outbreaks are usually related to contaminated food or water [17,18], while secondary transmission often results from person-to-person contact [19]. NoVs have also been identified in sporadic cases of gastroenteritis [20,21]. Although outbreaks and sporadic disease may occur year round, countries of the Northern hemisphere mainly show a seasonal pattern of increased occurrence during the winter months [22].

NoVs form a genus within the family of *Caliciviridae* and are genetically and antigenically highly variable. Five genogroups (G) of NoVs have been tentatively assigned from the molecular characterization of complete capsid gene sequences. Strains of three genogroups, GI, GII, and GIV, are found in humans (GII/11 are porcine), and GIII and GV strains are found in cows and mice, respectively [23].

Several large foodborne and waterborne outbreaks due to NoVs have been described [17,19,24]. However, there are only a few reports in which both epidemiological and environmental data have been confirmed by molecular data on the waterborne NoVs [25,26]. Up to 93% of the outbreaks and sporadic cases of nonbacterial, acute gastroenteritis in humans and about 60–85% of all gastroenteritis outbreaks, specifically within the United States, Europe, and Japan, are associated with NoVs [24].

In Greece, which has no surveillance system for non-bacterial gastroenteritis, the impact of NoVs infection is unknown, and very few epidemiological studies about NoVs have been reported so far. In fact, only two epidemiological studies have been reported previously in Greece [27,28] and only one refers to a possible waterborne outbreak [28]. Furthermore, there is no previous molecular epidemiological study in Greece about NoVs. The epidemiological as well as the molecular investigation of non-bacterial gastroenteritis is rather absent in Greece.

This report describes a large outbreak of non-bacterial gastroenteritis caused by NoVs in North Eastern Greece. It is the first study, to our knowledge in Greece, trying to combine environmental, epidemiological and molecular investigations in order to specify the cause and the conditions of the outbreak and to examine its characteristics. Also it is the first study which includes a molecular investigation of the NoVs identified during the studied outbreak.

## 2. Experimental Section

Xanthi is the capital of the prefecture of the same name located in North Eastern Greece. The population of Xanthi prefecture was 100;882 inhabitants, according to the National Census of 2001 [29]. Water supply of the capital and the main communities of the prefecture mainly depend on surface water sources, as well as on drilled wells. In the first days of June of 2006, reports of gastroenteritis cases to the General Hospital of Xanthi increased markedly.

### 2.1. Epidemiological Investigation

All gastroenteritis cases referred to the General Hospital of Xanthi during the period May 1, 2006 to November 5, 2006 (weeks 18–44) were ascertained using medical records from the Hospital. In addition, after cooperation with Health Office of the prefecture, a structured questionnaire was developed and used to gather clinical and sociodemographic information from in- and out-patients referred to the General Hospital of Xanthi the period between June 5 and September 3, 2006 (weeks 23–35). The questionnaire included information on gender, age, residence, symptoms, date of onset of symptoms, laboratory tests performed. Questionnaires were completed by health officers of the prefecture of Xanthi. An outbreak case was defined as any resident of Xanthi prefecture, who visited the hospital and having two or more episodes of vomiting and/or diarrhea, with/without abdominal pains and with/without laboratory confirmation during the period of June 5 and September 3.

In order to establish retrospective data for the epidemiological investigation, the gastroenteritis cases admitted to the paediatric and the Internal Medicine Unit from May till August of 2005 were also recorded.

### 2.2. Laboratory Investigation

One hundred and seventy four (174) stool specimens were collected from patients referred for acute gastroenteritis to the General Hospital of Xanthi from July 17 till September 3 (week 29–35). Specimens were analyzed for bacteria (*Salmonella, Shigella, Campylobacter*), parasites (*Cryptosporidium, Giardia*) and viruses (*Noroviruses*). A part of a specimen was stored at −20 °C for later testing for Norovirus. The rest was refrigerated and processed for bacteriological and parasitic examination within 12 hours after collection. Parasites (*Cryptosporidium* and *Giardia*) were detected by direct microscopy and *Salmonella, Shigella, Campylobacter, Staphylococcus* were sought by standard methods. For virological analysis, stool specimens were diluted 1:10 (w/v) with phosphate buffer saline (PBSa; Dulbecco’s formula) followed by thorough mixing and centrifugation at 3,000 × *g* for 5 min. Supernatants were stored at −20 °C until use. Viral RNA was extracted using QIAamp microspin columns (viral RNA mini kit; Qiagen, Crawley, UK) according to the manufacturer’s protocol and stored in aliquots at −80 °C. Purified RNA was used in an RT-PCR reaction according previously reported protocol which can detect GI and GII viruses [25]. The Qiagen One-Step RT-PCR kitTM was used throughout the study. A negative control containing water and a positive RT-PCR control containing RNA from one GI- and one GII-positive stool sample were included in each run. Two separate primer sets that amplify a 213-bp region of the RNA polymerase gene of Noroviruses for both genogroups were used [25]. A positive Norovirus was considered from the position on the gel (relative to the positive controls). Positive samples for Noroviruses were also confirmed by sequencing the amplified PCR product according to the previously reported method [25,30].

To determine the relatedness between different sequences of Greek NoVs strains (GR) detected in this study with GII sequences selected from GenBank database, MEGA 4.0.2 software [31] using the Neighbor-Joining method as well as Norovirus genotyping tool, Version 1.0 (http://www.rivm.nl/mpf/norovirus/typingtool) has been used [32]. Multiple sequence alignment of NoV sequences was performed using CLUSTALW2 software (www.ebi.ac.uk; data not shown).

### 2.3. Environmental Investigation

A concurrent investigation was carried out by the Office of Health of Xanthi and Department of Public Health of the University. An inspection and assessment of the hygienic situation of the areas where the main sources of water supply are installed was also carried out on 3^rd^ of July. Also, a water sample of one hundred liters (100 L) was collected from each one of the two main sources of surface water (river water used for the water supply of Xanthi prefecture) at July 10, 2006. As the cases were not decreasing, a second sampling from the same sites was performed on August 5, 2006. Briefly, the water samples were collected using an apparatus containing 1-MDS filter (Zetapore Virosorb, Cuno, USA) according to the previously described procedure [25]. The samples were eluted and further concentrated for Norovirus assay. The analysis for Norovirus detection was performed using reverse transcription-PCR (RT-PCR) as previously described [25].

### 2.4. Statistical Analysis

Data were analyzed using SPSS 17.0 (SPSS Inc., Chicago, IL, USA). Attack rate was calculated based on population data provided by the National Census of 2001 [29].

## 3. Results

### 3.1. Epidemiologic Investigation

**Figure 1.**
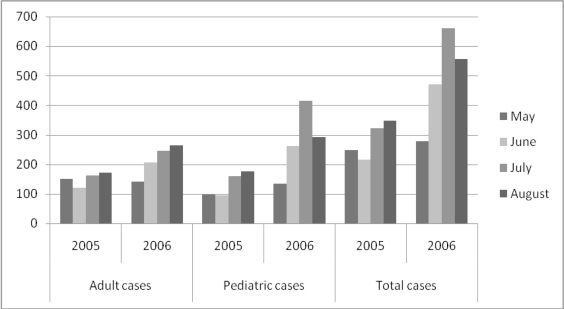
Gastroenteritis cases referred to the General Hospital of Xanthi (years 2005 and 2006). Adult cases: >15 y.o, Pediatric Cases: <15 y.o.

A total of 1,640 (45.2% male and 54.8% female, aged 3 months to 89 years) patients suffering from gastroenteritis were referred to the General Hospital of Xanthi from June 5 till September 3 (weeks 23–35), and were asked to answer the questionnaire. Of these cases, 72.1% reported diarrhoea, 82.4% reported vomiting, and only 12.5% reported fever. The average duration of illness was two days. 10.1% of the above patients were hospitalized at the Paediatric and the Internal Medicine units. In order to establish retrospective data for the epidemiological investigation, the gastroenteritis cases admitted to both units from May till August during 2005 and 2006 are listed (Figure 1).

**Figure 2.**
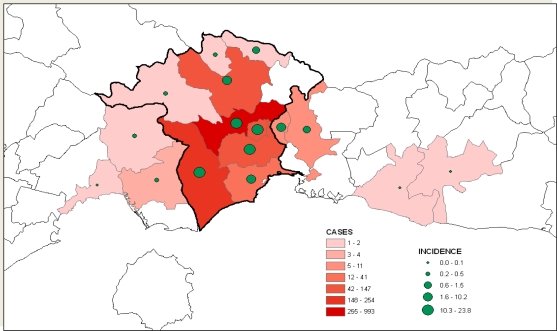
Incidence of gastroenteritis per municipality and municipal geographic part counted by the visits in General Hospital of Xanthi in the period between June 5 and September 3, 2006 (white regions represent areas outside the Xanthi Prefecture study area).

The number of gastroenteritis cases was similar to that of May 2006 and increased in June 2006 (p < 0.005, t-test). The area affected as well as the cases (and incidence rates) in the prefecture of Xanthi for the period of June 5 till September 3 is shown in Figure 2.

**Figure 3.**
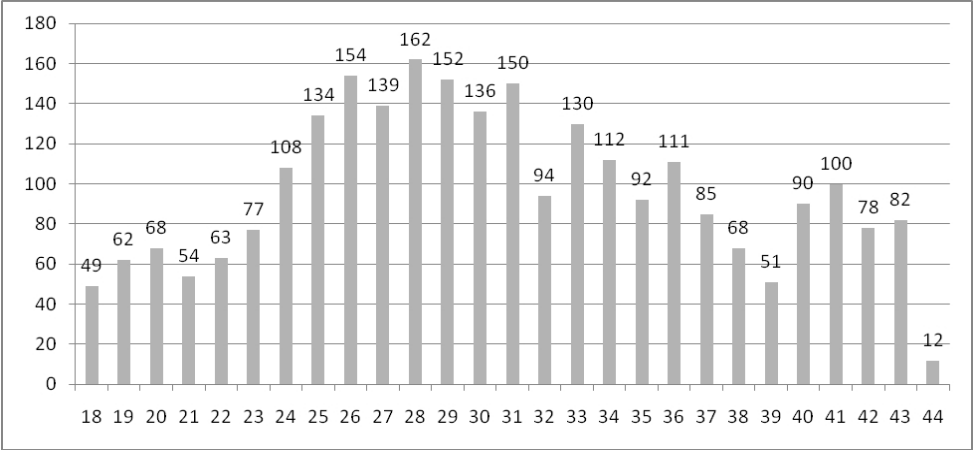
Number of gastroenteritis hospitalized cases per week in Xanthi’s hospital, during the period w18 to w44.

Figure 3 shows the weekly number of gastroenteritis cases admitted to hospital between May 1st and November 5th 2006 (weeks 18–44). The epidemic curve is based on week of reference to the hospital and shows a sharp increase of gastroenteritis cases during June, as well as high occurrence of cases is observed during July.

The overall attack rate for gastroenteritis for the period June 5 till September 3 (weeks 23–35) was 16.3 cases/1,000 inhabitants. The age-specific attack rates are shown in Table 1. About 57% of cases were observed under the age of 15 years. There were no considerable differences in the age and sex distribution of the cases over the course of the outbreak.

### 3.2. Laboratory Investigation

Faecal specimens were obtained from 174 in- and out-patients referred to the General Hospital of Xanthi (10.6% of total cases). Faecal samples were collected over the period of 17th of July and 3rd of September (weeks 29–35). Six fecal specimens (3.4%) were found positive for *Salmonella typhimurium* (2), *Campylobacter* (1), *Cryptosporidium parvum* (2), *Giardia lamblia* (2). Ninety nine of them (56.8%) resulted positive for Noroviruses. All the positive specimens were characterized as NoVs GII. Furthermore, twenty-two (22) PCR products (12.64%) were confirmed by sequencing. Sequence BLAST search and Norovirus genotyping tool, showed that these outbreak isolates were GII strains presenting a homology of average 94.8% (86–97%) to GII strain obtained from GenBank (EF621480 GII.4 strain).

### 3.3. Environmental Investigation

All water samples were tested negative for Noroviruses. Water samples were also examined microbiologically (*E.coli*, fecal coliforms and *Enterococci*) and chemically (nitrate) and their quality were assessed to be acceptable according to the national water quality guidelines. The water treatment method was only non-stable chlorination. The sanitary inspection of the drinking water sources revealed a low level of hygienic conditions (e.g., dirty areas around the sources, unprotected sources, *etc*.). A few local communities were installed close to the two main drill points of the water supply (which were interconnected) and these communities did not fulfill elementary hygienic conditions such as chlorination or protection of the drills. In addition, a heavy rainfall (>250 mm) had been recorded during the first five days of June.

## 4. Discussion

The epidemic curve presented a rapid increase of gastroenteritis cases during June, as well as high occurrence of cases is observed during July. The number of cases declined slowly during the next three months and the epidemic finally stopped during the first days of November. This epidemic curve suggests an occurrence of an outbreak and its beginning related to a possible common source of contamination. The initial source could have been contaminated drinking water, caused by the poor hygienic conditions of the closeby local communities and the intense rainfall that preceded the emergence of the outbreak. The environmental and hygienic conditions in the areas where water supply sources are installed, suggest an increased risk of drinking water contamination, although no analytical epidemiological study was performed to confirm the waterborne source of the outbreak. Although the water samples tested did not confirm this suggestion and no NoVs were detected, we have to clarify that these samples were taken after a significant time delay. The delay of the water sampling was due to delayed announcement of the outbreak by the water company as well as the lack of a surveillance system for non-bacterial gastroenteritis. Therefore, the negative results of the environmental investigation do not exclude the possibility of a waterborne emergence of the outbreak. The waterborne hypothesis is supported by the fact that the outbreak studied is the second gastroenteritis epidemic reported in almost the same area [28]. A possible waterborne gastroenteritis outbreak due to Norovirus infection in Xanthi during the period January 28 to February 10, 2005 had been previously described. Papadopoulos *et al.* stated that for the most suspicious event that could have been responsible for the contamination of a main well and probably responsible for the NoVs outbreak was a water flood which resulted from heavy rain during the night of January 27, 2005 [28]. During this outbreak, a total of 709 patients visited the local hospital over a period of two weeks with symptoms of acute gastroenteritis. This is the only Norovirus outbreak reported in the literature in Greece [28]. The occurrence of two large outbreaks of gastroenteritis in the same area within two years after a similar event (heavy rainfall) suggests a low level of sanitary conditions and a possible source of repeated contamination of drinking water.

As observed in other outbreaks, this outbreak affected all age groups [33–36]. Cases represented all ages (range 0.25–89 y.o). However, young people presented higher attack rates, this could have been related to a lower level of natural immunity, to frequent contacts among children or to the fact that people tend to go to the hospital more quickly for their children. In Greece, there is no reporting surveillance system for gastroenteritis outbreaks, and it is estimated that consequently a large number of gastroenteritis cases are not recorded and as a result, many cases of gastroenteritis that occurred during the outbreak may have not been recorded. This could be a fact mainly for mild cases or cases occurring in rural areas. A number of patients probably contacted private physicians, or rural Health Centers existing in the studied area. Such patients were not recorded as part of the cases of the studied outbreak. These methodological issues concerning case identification and case ascertainment put some limitations to the interpretation of our data. However, there are strong indications for the outbreak such as that the epidemic curve showed a fast increase of cases at the emergence and the duration of the outbreak was many times longer than the incubation period of noroviruses (12–48 hours).

Sequence BLAST search and the Norovirus genotyping tool of Greek NoVs strains’ sequences along with reference GII strains confirmed that the outbreak isolates belonged to GII but the identification of GII genotypes was not possible due to the short amplified sequence of the RNA polymerase gene. While the RNA polymerase region is mostly used for diagnosis of NoVs, a complete identification requires the use of the capsid region [30] which was not possible in our study (due to the high cost). The use of short sequences has been successful for establishing diagnoses of NoV infection, but it becomes problematic for the classification or phylogenetic analysis [23]. Our results show that the outbreak was caused by GII strains. This is in accordance with studies from different parts of the world reporting the predominance of GII [6,16,21,36,37].

Outbreaks in hospitals and institutions are more easily identified, cause more disruption compared to outbreaks in the community and are more likely to be reported [38]. Also the community outbreaks are more difficult to yield clinical specimens for identification of Norovirus. The emerging behavior of Norovirus has stimulated a considerable interest in the molecular epidemiology of these viruses. In turn, this has been complicated by the underestimated rate of recombination between strains [35,36] and the lack of a simple *in vitro* culture system with high yield.

## 5. Conclusions

The outbreak is one of the very few extended outbreaks due to Noroviruses in Greece. Due to the lack of a surveillance system for acute gastroenteritis in Greece, the accomplishment of the study proved very difficult. The emergence of the outbreak was possibly due to contaminated drinking water, although this was not proved by water analyses data. Its long duration is likely due to a large number of cases related to person-to-person contamination. The fact that the described outbreak was the second gastroenteritis outbreak in the same area, within the time interval of two years, suggests prolonged negligence in drinking water sanitary conditions (e.g., lack of stable chlorination). Also, the present epidemiological study confirmed the necessity of the development of a surveillance system for acute non-bacterial gastroenteritis outbreaks in Greece.

## Figures and Tables

**Table 1.** Age-specific Attack Rates (w23–w35).

**Age-groups (y.o)**	**Number of Cases**	**Population**	**Attack Rates/1000 inhabitants**
<1	121	1,286	94.1
1–4	495	5,147	96.2
5–14	342	13,055	26.2
15–24	183	16,535	11.1
25–44	210	29,650	7.1
45–64	146	22,309	6.5
≥65	143	12,900	11.1
Total	1640	100,882	16.3
